# Effect of Oxidized LDL on Platelet Shape, Spreading, and Migration Investigated with Deep Learning Platelet Morphometry

**DOI:** 10.3390/cells10112932

**Published:** 2021-10-28

**Authors:** Jan Seifert, Hendrik von Eysmondt, Madhumita Chatterjee, Meinrad Gawaz, Tilman E. Schäffer

**Affiliations:** 1 Institute of Applied Physics, University of Tübingen, 72076 Tübingen, Germany; jan.seifert@uni-tuebingen.de (J.S.); hendrik.von-eysmondt@uni-tuebingen.de (H.v.E.); 2 Department of Cardiology and Angiology, University of Tübingen, 72076 Tübingen, Germany; madhumita.chatterjee@med.uni-tuebingen.de (M.C.); meinrad.gawaz@med.uni-tuebingen.de (M.G.)

**Keywords:** platelet, deep learning, neural network, oxLDL, platelet shape, platelet migration, atherosclerosis, lipid

## Abstract

Platelets are functionally versatile blood cells involved in thrombosis, hemostasis, atherosclerosis, and immune response. Platelet interaction with the immediate microenvironment in blood, vasculature, and tissues alters platelet morphology. The quantification of platelet morphodynamics by geometrical parameters (morphometry) can provide important insights into how platelets sense and respond to stimulatory cues in their vicinity. However, the extraction of platelet shapes from phase contrast microscopy images by conventional image processing is difficult. Here, we used a convolutional neural network (CNN) to develop a deep-learning-based approach for the unbiased extraction of information on platelet morphodynamics by phase contrast microscopy. We then investigated the effect of normal and oxidized low-density lipoproteins (LDL, oxLDL) on platelet morphodynamics, spreading, and haptotactic migration. Exposure of platelets to oxLDL led to a decreased spreading area and rate on fibrinogen, accompanied by increased formation of filopodia and impaired formation of lamellipodia. Haptotactic platelet migration was affected by both LDL and oxLDL in terms of decreased migration velocity and reduced directional persistence. Our results demonstrate the use of deep learning in investigating platelet morphodynamics and reveal differential effects of LDL and oxLDL on platelet morphology and platelet–matrix interaction.

## 1. Introduction

Platelets circulate in the blood in a resting state and constantly monitor changes in vasculature [1]. They actively engage in hemostasis, wound healing, and immune response [2] upon detecting vascular injury, inflammation, or changes inflicted by pathogenic intrusion. Activated platelets adhere and spread at the site of vessel injury and aggregate to form a blood clot, thereby closing the damaged vessel wall [3,4]. During spreading, the platelet-covered area increases owing to rapidly formed filopodia and lamellipodia, and the platelet shape changes from a discoid to a flat, fully spread shape [5,6,7]. Platelet migration in response to chemotactic cues of pathogenic or host origin has been studied for many years [8,9,10,11,12]. Recently, it has been shown that platelets contribute to innate immune response by haptotactic migration [13,14,15]. Platelet morphodynamics during spreading and migration are therefore significant characteristics of platelet interaction with the immediate microenvironment, and the quantification of morphodynamics in terms of morphometric parameters is of high relevance.

A wide variety of techniques have been used to investigate platelet shape, including optical brightfield [6,16] and fluorescence microscopy [7], electron microscopy [17,18], and scanning probe microscopy [16,19,20,21,22,23]. Many of these approaches have drawbacks such as extensive preparation, additional staining steps, and limited throughput, or require complex analysis. Deep learning neural networks, benefiting from hugely increased computational power in recent years, can help to overcome these limitations [24,25,26,27,28]. In hematology, neural networks have been used for blood cell [29,30] and platelet classification [31].

The primary objective of this investigation was to devise an approach for studying platelet morphodynamics with optical phase contrast microscopy in combination with U-net, a deep learning convolutional neural network (CNN) developed for cell segmentation [32]. The CNN is used to binarize phase contrast images of platelets and to separate platelets from the background, which is a key step for automated shape analysis. We used images of membrane-stained platelets to semi-automatically generate a large training pool of images for neural network training. With the trained CNN, it was possible to analyze platelet shape following a variety of treatments and to investigate time-resolved platelet morphodynamics during spreading and migration.

We used our deep learning morphometry to investigate the influence of lipoproteins, which are transport proteins for fat/lipid molecules in the blood, on platelet function. Increased blood levels of low-density lipoproteins (LDL) are a risk factor causing atherothrombosis and coronary artery disease [33,34,35]. Both LDL and oxidized LDL (oxLDL) are accumulated at the fatty streaks, initiating the formation of an atherosclerotic plaque under hyperlipidemic conditions, and have distinct prothrombotic effects inducing platelet activation. Both LDL and oxLDL activate platelets through overlapping and distinctive pathways [33,36] downstream of their respective receptors, e.g., ApoER2 for LDL [37] and CD36, LOX-1, or transmembrane-CXCL16-SR-PS/Ox for oxLDL [38]. OxLDL-mediated platelet activation through CD36 has been investigated by many groups [39,40,41]. We previously showed a dose-dependent effect of LDL and oxLDL on platelet activation, leading to platelet degranulation, CD62P surface exposure, αIIbβ3-integrin activation, and platelet adhesion, spreading and aggregation [42]. Moreover, both LDL and oxLDL increase thrombus formation ex vivo, while the administration of LDL and oxLDL in mice triggers thrombus formation in the injured carotid artery in vivo [42,43,44]. Both LDL and oxLDL can influence the redox status of platelets, prompting the reactive oxygen species (ROS)-mediated oxidation of LDL in the platelet microenvironment and intraplatelet LDL-to-oxLDL conversion in activated platelets. These oxidative functions involve the active participation of NADPH oxidase 2 (NOX2) [45], mitochondrial superoxide generation [42], and significant intraplatelet ROS formation over the basal state [42]. Capitalizing on such previous investigations from our group and others, the current investigation was designed to explore platelet morphodynamics under the influence of LDL and oxLDL, showing that the treatment of platelets with oxLDL induced the formation of filopodia and retraction of lamellipodia, led to a decreased spreading area and rate on fibrinogen, and reduced the ability for haptotactic migration. Employing a novel deep learning approach, we devised a high-throughput, automated, and unbiased image analysis method. Our results suggest that platelet activation by LDL and oxLDL can influence the interaction with biological matrices and platelet functions.

## 2. Methods

### 2.1. Platelet Isolation and Serum Preparation

Washed human platelets were isolated from freshly drawn blood of healthy volunteers mixed with acid citrate dextrose (at a ratio of 1:7) to prevent coagulation. Platelet-rich plasma (PRP) was obtained from whole blood by centrifugation at 200× *g* for 20 min. Tyrode-HEPES buffer (136.89 mM NaCl, 2.81 mM KCl, 11.9 mM NaHCO_3_, 1 g/L D-glucose, 10 mM HEPES), pH adjusted to 6.5 with HCl, was added to the PRP at a ratio of 3:1. Then, washed platelets were isolated by centrifugation at 880× *g* for 10 min and careful resuspension of the platelet pellet in Tyrode-HEPES buffer, pH 7.4.

For platelet migration experiments, human serum was prepared from coagulated blood in serum monovettes (02.1063.001, Sarstedt, Nümbrecht, Germany). After phlebotomy, blood was allowed to coagulate for 30 min. Then, serum was collected from the coagulated blood by two centrifugation steps at 2000× *g* for 15 min each.

### 2.2. Platelet Activation and Treatment

For platelet spreading experiments, platelets were activated by 0.1 U/mL thrombin (T6884, Sigma Aldrich, St. Louis, MO, USA), 20 µg/mL oxLDL (770252, Kalen Biomedical, Germantown, MD, USA), 20 µg/mL LDL (770200, Kalen Biomedical), or were left untreated before spreading onto fibrinogen-coated (F3879, Sigma Aldrich) glass-bottom dishes (81218, ibidi, Gräfeling, Germany) in Tyrode-HEPES buffer, pH 7.4. From our previous investigation it was established that 20 µg/mL oxLDL or 20 µg/mL LDL promotes significant platelet activation and spreading [42]; we therefore continued to employ this concentration for the present study. Platelets were pretreated for 10 min with oxLDL before the experiments. For NOX2 inhibition experiments, platelets were incubated with 1 mM apocynin (178385, Sigma Aldrich) prior to treatment with oxLDL (Supplementary Figure S4c). Unless indicated otherwise, 1 mM calcium was added to the Tyrode-HEPES buffer used for the experiments.

For platelet migration experiments, glass-bottom dishes (81218, ibidi) were coated for 5 min with migration buffer consisting of Tyrode-HEPES buffer, pH 7.4, 0.1 mg/mL fibrinogen (F3879, Sigma Aldrich), 3 µM U-46619 thromboxane (TXa, Cay16450, biomol, Hamburg, Germany), 20 µM adenosine diphosphate (ADP, A2754, Sigma Aldrich), and 5% human serum. Afterwards, washed platelets were activated for 1 min in migration buffer, added to the coated glass-bottom dishes, and were allowed to adhere and spread for 10 min. Then, non-adherent platelets were carefully washed out and migrating platelets were imaged at 37 °C after 30 min. For platelet migration experiments in the presence of LDL or oxLDL, platelets were treated with 20 µg/mL LDL or 20 µg/mL oxLDL in migration buffer for 10 min before addition to the glass-bottom dish containing migration buffer with LDL or oxLDL. As a control, non-migrating platelets were prepared using migration buffer without human serum and oxLDL.

### 2.3. Microscopy and Acquisition of Training Data

Phase contrast and fluorescence images were recorded using an inverted microscope (Ti-E, Nikon, Tokyo, Japan) equipped with a digital camera (Qi-2, Nikon), a stage-top incubator (10722, ibidi) and a 20× air and 100× oil immersion objective. Time-lapse imaging was performed by recording phase contrast images every 30 s for migration experiments and every 10 s for all other experiments.

For the acquisition of training data, washed platelets were stained with 2.5 µM CellMask Orange (C10045, ThermoFisher, Waltham, Massachusetts, USA) for 10 min at 37 °C. The stained platelets were then added to a fibrinogen-coated (0.1 mg/mL) glass-bottom Petri dish (81218, ibidi) containing Tyrode-HEPES buffer and 0.1 U/mL thrombin (T6884, Sigma Aldrich). Then, simultaneous phase contrast and fluorescence images were recorded while platelets adhered and spread. The fluorescence images were processed by a rolling-ball background removal algorithm to remove blurry edges for better edge detection. Afterwards, binary images were created from the processed fluorescence images by applying a threshold and filling the remaining holes (Supplementary Figure S1). The resulting ground truth images were examined by eye, and errors were corrected manually.

### 2.4. Training of the U-Net and Network Predictions

The neural network architecture of the U-net was implemented in TensorFlow (v. 1.12) with the Python deep learning API Keras (v. 2.2.4). The code for neural network training and prediction tasks was written in Python (v. 3.6). The U-net architecture was implemented for a resolution of 2048 × 2048 pixels to fully utilize the native resolution of the digital camera (1608 × 1608). All images were resized by edge reflection padding for the use as network input, and the outputs of the network were resized to the native resolution by cropping. For both magnifications (100× and 20×), separate networks were trained. The image sets used for network training contained *n* = 170 and *n* = 82 phase contrast and ground truth image pairs with a total of *n* = 2064 and *n* = 12,739 platelets for the 100× and 20× magnifications, respectively. For network training, phase contrast images were normalized by $I^{\prime }\left(x,y\right)=\frac{I\left(x,y\right)-I_{\min}}{I_{\max}-I_{\min}}$ with minimum and maximum intensities *I*_min_ and *I*_max_. Data augmentation (rotation, flipping, and resizing) was used and the network was trained until the validation loss coefficient reached a minimum level. The training tasks were performed at the computational center bwForCluster BinAC on a graphics processing unit (Tesla K80, Nvidia). The total training duration was 80 h for the 100× and 40 h for the 20× magnification. Predictions of the trained networks were computed in Python on a personal computer using padding and normalization as described above.

### 2.5. Calculation of Shape Parameters

Platelet shape analysis was completed in the analysis software Igor Pro (WaveMetrics, Portland, OR, USA). The position coordinates and platelet area *A*, circularity ($c=4\pi A/P^{2}$, with outline perimeter *P*), and aspect ratio (e=a/b, with long and short axes *a* and *b* of an ellipse fit to the platelet outline) were calculated using a built-in function. The filopodia-counting algorithm using length and curvature constraints to determine the endpoints of individual filopodia was adapted from Sandmann et al. [7]. The platelet curvature was calculated by point-wise determination of the curvature of the platelet outline, generated with a contour-tracking algorithm [46]. Platelet spreading was analyzed by calculating idle time (from adhesion to 15% area increase) and initial spreading rate (during the 2 min following the end of idle time). The position of migrating platelets was tracked as the centers of the platelet outlines in subsequent images of an image sequence. Platelets that could not be tracked over the whole sequence were excluded from analysis. The migration velocity was calculated as the accumulated distance divided by time. The straightness of the migrated path was calculated as the Euclidean distance divided by the accumulated distance. The directional change in a given time interval was calculated as the cosine of the angle *θ* between the directions at the start and end of the analyzed time interval; the average was then determined by averaging over all available time intervals.

### 2.6. Statistics

Data were analyzed and processed in Igor Pro (WaveMetrics). Data are presented as medians ± quartiles unless stated otherwise. All results were tested using Dunn’s test for non-parametric multiple comparisons. Results were considered significantly different for *p*-values < 0.001 (***).

## 3. Results

### 3.1. Platelet Shape Analysis with U-Net

**Figure 1:**
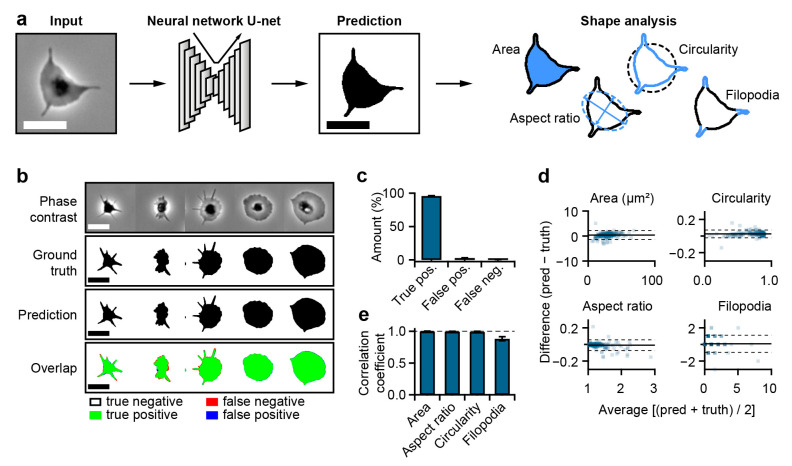
Platelet detection by convolutional neural network (CNN) and evaluation of platelet shape parameters. (**a**) Workflow for CNN-supported shape analysis. (**b**) Comparison of ground truth and network prediction for platelets of different shapes. (**c**) Amount of true positive, false positive, and false negative pixels (sum of all three is 100%). (**d**) Bland–Altman plots for platelet shape parameters gained from the prediction and ground truth images. The solid and dashed lines indicate the mean ± 1.96 × standard deviation of the difference between prediction and ground truth shape parameters. (**e**) Pearson’s correlation coefficient *R* for the shape parameters in panel d. Scale bars: 5 µm.

We used U-net, a deep learning CNN developed for cell image segmentation [32], for the binarization of phase contrast images into two different regions (Figure 1a): platelets (black) and non-platelet background (white). These binary images were then used for subsequent shape analysis. For training and validation, we recorded phase contrast and fluorescence images of membrane-stained platelets. From the fluorescence images, we generated binary masks (separating platelets from the background), which served as the ground truth for the CNN for each phase contrast image (Supplementary Figure S1a). For validation of the trained network (Supplementary Figure S1b), we examined phase contrast images not used for training. Platelets of different shapes were recognized by the CNN with a good match of the prediction with the ground truth (Figure 1b). A pixel-wise comparison of prediction and ground truth indicated a high ratio of true positive pixels (96% intersection over union) (Figure 1c). Subsequently, we quantified the platelet shapes in terms of platelet area, aspect ratio (length/width), circularity (measure of “roundness”; value of 1: perfect circle), and number of filopodia from the ground truth and from the prediction (Figure 1a). The prediction and ground truth shape parameters (*n* = 226 platelets) were in excellent agreement, as depicted by the Bland–Altman plots showing the difference of prediction and ground truth parameters plotted against the average for each single platelet (Figure 1d; solid and dashed lines indicate the mean ± 1.96 × standard deviation). This excellent agreement goes hand-in-hand with a strong correlation between the prediction and ground truth shape parameters (Figure 1e). We also trained a separate CNN using lower-magnification images, which allowed for the determination of platelet area and aspect ratio with decent accuracy (Supplementary Figure S2), thereby increasing the throughput in platelet dynamics measurements.

### 3.2. OxLDL Influences Platelet Shape and Interaction with Matrix

**Figure 2:**
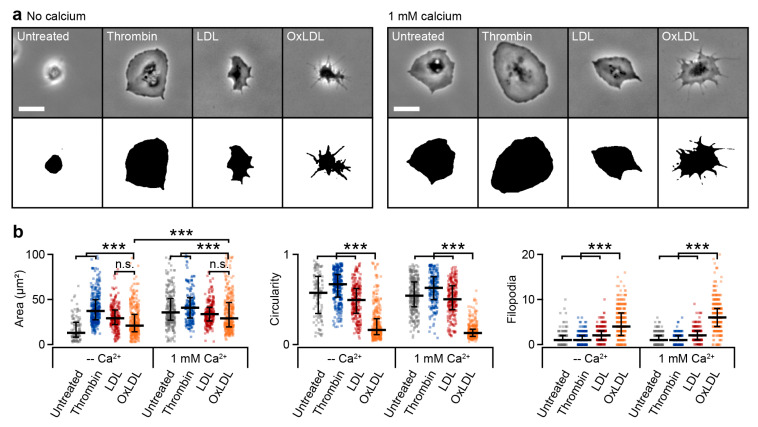
Oxidized low-density lipoprotein (oxLDL) influences platelet shape. (**a**) Phase contrast images and predictions and (**b**) shape parameters of untreated, thrombin-treated (0.1 U/mL), LDL-treated (20 µg/mL), and oxLDL-treated (20 µg/mL) platelets after spreading on fibrinogen for 30 min. Platelet numbers (no calcium, calcium): Fibrinogen: *n* = (113, 285), thrombin: *n* = (315, 194) (thrombin), LDL: *n* = (240, 248), oxLDL: *n* = (395, 342). *** indicates statistically significant difference (*p* < 0.001). Scale bars: 5 µm.

Previously, we showed that LDL and oxLDL trigger αIIbβ3–integrin activation and PAC-1 binding [42]. We therefore investigated the interaction of oxLDL-treated platelets with fibrinogen. As a comparison, platelets were left untreated or were treated with thrombin or LDL. OxLDL-treated platelets exhibited an irregular, dendritic shape as compared to untreated, thrombin-treated, or LDL-treated platelets (Figure 2a, Supplementary Figure S3). OxLDL-treated platelets were able to adhere to fibrinogen in the absence of extracellular calcium, but they were significantly less spread than in the presence of calcium (Figure 2a). In the presence of calcium, oxLDL-treated platelets had a significantly lower spreading area, decreased circularity, and increased number of filopodia compared to untreated, thrombin-treated, and LDL-treated platelets (Figure 2b). Inhibition of NADPH oxidase by apocynin [47] led to a decrease in spreading area in oxLDL-treated platelets; oxLDL-induced changes in platelet circularity or number of filopodia remained unaffected (Supplementary Figure S4).

**Figure 3:**
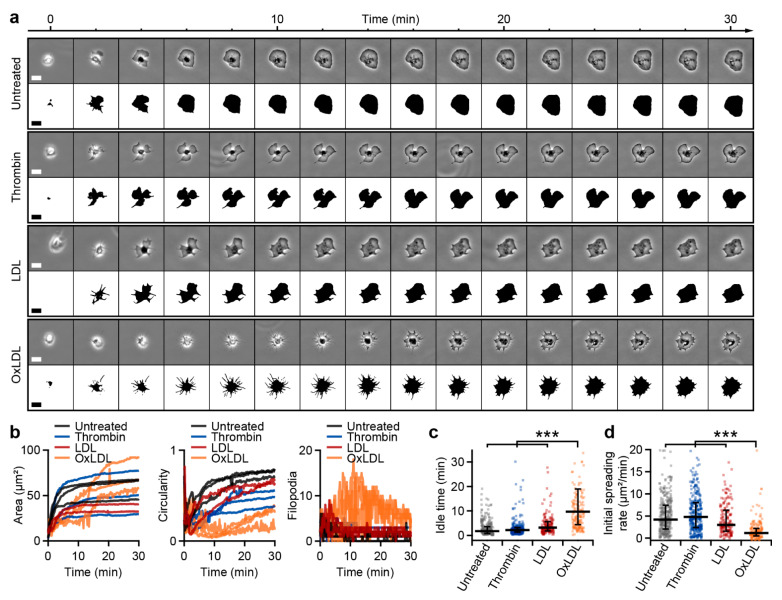
OxLDL influences platelet spreading. (**a**) Phase contrast image sequence and predictions of untreated, thrombin-treated, and oxLDL-treated platelets during spreading on fibrinogen (see also Supplementary Video S1). (**b**) Shape parameters as a function of time for three exemplary untreated, thrombin-, LDL-, and oxLDL-treated platelets each. Timepoint of adhesion is at *t* = 0 min. (**c**) Idle time (time from adhesion to 15% area increase) and (**d**) initial spreading rate (area increase during the 2 min following the end of idle time) for *n* = 322 untreated, *n* = 282 thrombin-treated, *n* = 161 LDL-treated, and *n* = 173 oxLDL-treated platelets. *** indicates statistically significant difference (*p* < 0.001). Scale bars: 5 µm.

Next, we investigated the spreading dynamics of oxLDL-treated platelets in the presence of calcium. Thrombin-treated platelets typically reached a spread-out shape within 5 min after contact with a fibrinogen-coated surface (Figure 3a, Supplementary Video S1). OxLDL-treated platelets had a dendritic and slowly extending shape with many filopodia during spreading. Analysis of the platelet area revealed two phases of area increase for untreated, thrombin-treated, and LDL-treated platelets (Figure 3b, black, blue and red traces): a phase of fast area increase during the first 5 min after adhesion, followed by a phase of slower area increase. The first phase was dominated by the formation of filopodia, which were subsequently retracted during the second phase, leading to an increase in circularity (Figure 3b, black, blue, and red traces). For oxLDL-treated platelets, the initiation of spreading was delayed, and a phase of rapid area increase was not observed (Figure 3b, orange traces). The spreading area increased slowly with a filopodia-dominated shape and low circularity. OxLDL-treated platelets showed a significantly increased idle time (Figure 3c) and a significantly decreased initial spreading rate compared to untreated, thrombin-treated, and LDL-treated platelets (Figure 3d).

### 3.3. OxLDL Induces Formation of Filopodia and Retraction of Lamellipodia

**Figure 4:**
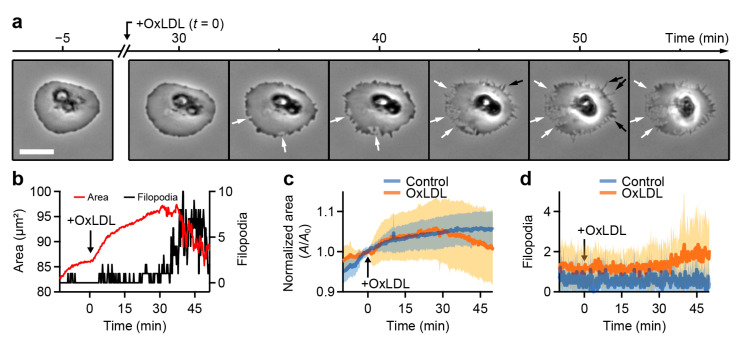
OxLDL induces formation of filopodia and retraction of lamellipodia. (**a**) Image sequence of a spread platelet during the addition of oxLDL (20 µg/mL) showing the retraction of lamellipodia (white arrows) and the formation of filopodia (black arrows). Platelets were activated with thrombin (0.1 U/mL) prior to spreading to the fibrinogen surface. See also Supplementary Video S2 and Supplementary Figure S5a for the corresponding predictions. (**b**) Area and filopodia of the platelet shown in (a) as a function of time. (**c**) Normalized area (*A*_0_: area at the time of addition of oxLDL) and (**d**) number of filopodia of platelets during the addition of oxLDL (control: addition of PBS). The solid lines and shaded areas indicate mean ± standard deviation. Scale bar: 5 µm.

We examined the effect of oxLDL on filopodia and lamellipodia formation in platelets spread on fibrinogen. After the addition of oxLDL, the platelet edges started to become ruffled (Figure 4a), followed by a retraction of the lamellipodium (white arrows) and the formation of filopodia (black arrows) (see also Supplementary Video S2). The platelet showed a reduction in spreading area and a simultaneous increase in the number of filopodia starting at ≈30 min after the addition of oxLDL (Figure 4b). The same results were obtained for the averages of many platelets (Figure 4c,d; *n* = 14 for oxLDL-treated and *n* = 9 for control platelets).

### 3.4. OxLDL Impairs Haptotactic Platelet Migration

**Figure 5:**
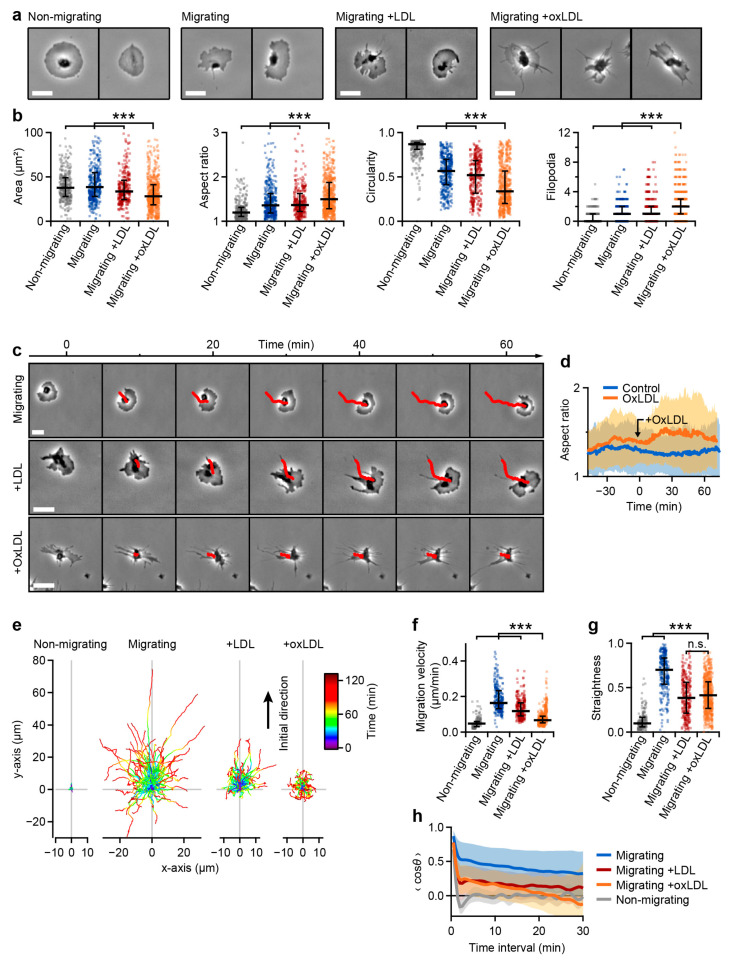
OxLDL alters the dynamics of haptotactic platelet migration. (**a**) Images and (**b**) shape parameters of *n* = 319 non-migrating, *n* = 384 migrating, *n* = 305 LDL-treated (20 µg/mL), and *n* = 632 oxLDL-treated (20 µg/mL) migrating platelets. For the corresponding predictions see Supplementary Figure S5b. (**c**) Image sequence of untreated, LDL-treated (20 µg/mL), and oxLDL-treated (20 µg/mL) migrating platelets (see also Supplementary Video S3). (**d**) Aspect ratio of migrating platelets as a function of time with and without (control) the addition of oxLDL. The solid lines and shaded areas indicate mean ± standard deviation. (**e**) Migration paths of non-migrating, untreated, LDL-treated, and oxLDL-treated migrating platelets. The initial direction of migration was aligned along the positive y-axis. (**f**) Migration velocities of *n* = 169 non-migrating, *n* = 246 untreated, *n* = 264 LDL-treated, and *n* = 590 oxLDL-treated migrating platelets. (**g**) Straightness of the migrated paths (Euclidean distance / accumulated distance). A value of 1 indicates a perfectly straight movement. (**h**) Average directional change as a function of time, quantified by the average cosine of the angle *θ* between the directions at the start and at the end of the analyzed time interval (straight movement: <cos*θ*> = 1, random movement: <cos*θ*> = 0). The solid lines and shaded areas indicate mean ± standard deviation. *** indicates statistically significant difference (*p* < 0.001). Scale bars: 5 µm.

We further investigated platelets in a microenvironment favoring haptotactic migration (Figure 5). Migrating platelets showed typical indicators of migration: an elongated, polarized shape with a ruffled membrane (Figure 5a) [13]. OxLDL-treated migrating platelets had a similar appearance but with prominent filopodia (Figure 5a, right), a significantly decreased spreading area, increased aspect ratio, decreased circularity, and increased number of filopodia (Figure 5b). Despite the different shape, oxLDL-treated platelets were able to migrate (Figure 5c, Supplementary Video S3). Addition of oxLDL to untreated migrating platelets increased their aspect ratio within the first 30 min after addition (Figure 5d)—this timescale is similar to the retraction of lamellipodia described in Figure 4.

Untreated migrating platelets covered a Euclidean distance of up to 80 µm within 120 min and mostly followed their initial direction (aligned in +*y* direction, Figure 5e). During the same time, both LDL- and oxLDL-treated platelets covered a smaller Euclidean distance (up to 30 µm and 10 µm, respectively). The migration velocity (migrated distance divided by time) for oxLDL-treated platelets was smaller than for untreated or LDL-treated migrating platelets, but still larger than for non-migrating platelets (Figure 5f). Additionally, oxLDL- and LDL-treated migrating platelets followed a less straight migration path (Figure 5g) and were more likely to change their direction (Figure 5h).

## 4. Discussion

We developed a neural-network-based approach for the binarization of platelet phase contrast images with subsequent shape analysis (Figure 1) using the deep learning CNN U-net, which was introduced by Ronneberger et al., and which has been applied to cell segmentation, counting, and morphometry in microscopy and medical imaging [32,48]. The binarization of microscopy images, that is, the separation of cells from the background, is a key step for cell shape analysis. Our approach allows for a fast and unbiased binarization that is independent of filters, thresholds, or manual masking. The predictions of the CNN and the extracted shape parameters showed an excellent agreement and strong correlation with the ground truth (Figure 1), consistent with previous applications of U-net on cells [32].

We used our deep learning neural network approach to investigate the effect of oxLDL on platelet morphodynamics during spreading and migration with high-resolution and high-throughput readout. The spreading of platelets on fibrinogen was calcium-dependent [49,50], and oxLDL-treated platelets showed an irregular dendritic shape during spreading with an increased formation of filopodia (Figure 2 and Figure 3), which is indicative of significant platelet activation in early stages of spreading [7]. The scavenging of ROS by the NADPH oxidase inhibitor apocynin led to a reduced spreading area of oxLDL-treated platelets, showing the significance of intracellular ROS generation for oxLDL-induced platelet activation. Furthermore, oxLDL led to a decreased spreading rate on fibrinogen. Unlike untreated, thrombin-treated, or LDL-treated platelets, which have two distinct phases of fast and slow spreading [6], oxLDL-treated platelets showed a slowly, gradually increasing area during spreading, accompanied by the formation of filopodia. In spread platelets, oxLDL induced the retraction of lamellipodia (Figure 4). The interplay of lamellipodia and filopodia formation, which is crucial for complete spreading on fibrinogen [7], seems to be impaired in oxLDL-treated platelets. OxLDL is known to activate platelets and lead to platelet aggregation and thrombus formation [41,42,43,44], depending on the degree of oxidation [51,52]. An increased formation of filopodia in oxLDL-treated platelets has been observed in previous work [42]. Lamellipodia are not crucial for aggregation and thrombus formation [53,54], but do affect platelet migration [15]. An impairing effect of oxLDL on the formation of lamellipodia has also been shown for macrophages, where it leads to a loss of cell polarity and locomotion [55] and increased formation of filopodia by increased actin polymerization [56]. In our experiments, the ability of platelets for haptotactic migration was not completely inhibited by oxLDL, but the migration velocity and the directional persistence were reduced. Our observations suggest that platelet activation with oxLDL influences platelet interaction with fibrinogen and haptotactic migration. Such LDL- and oxLDL-driven changes are executed through respective receptors (e.g., ApoER2 for LDL and CD36, LOX-1, or CXCL16-SR-PS/Ox for oxLDL). A thorough, in-depth investigation is called for to ascertain the relative contribution of these receptors to LDL- and oxLDL-driven alterations in platelet morphodynamics by employing pharmacological receptor antagonists or mouse lines genetically deficient in these receptors. Moreover, as hyperlipidemia is a prominent cardiovascular risk factor, morphodynamic analysis of platelets from obese and hyperlipidemic patients as compared to age-matched normolipidemic subjects would potentially reveal the clinical significance of the current explorative observations.

## Data Availability

The data used and analyzed for this study are available from the corresponding author upon reasonable request.

## Supplementary Materials

The following are available online at https://www.mdpi.com/article/10.3390/cells10112932/s1, Figure S1: Training of the CNN, Figure S2: Comparison of 100× and 20× magnification, Figure S3: Impact of extracellular calcium on platelet spreading, Figure S4: Platelet shape and ROS generation, Figure S5: CNN predictions, Video S1: Platelets with different treatments during spreading, Video S2: OxLDL-induced retraction of platelet lamellipodia, Video S3: Platelets with different treatments during haptotactic migration.
