# Supplementary material for: Effect of Oxidized LDL on Platelet Shape, Spreading, and Migration Investigated with Deep Learning Platelet Morphometry
Source: Cells. 2021 Oct 28;10(11):2932. doi: 10.3390/cells10112932 (PMC8616354; doi:10.3390/cells10112932)
Supplement: Supplementary file 1 [file cells-10-02932-s001.zip › cells-platelets-neural-network-supplementary.pdf]

Supplementary information for:

Effect of oxidized LDL on Platelet Shape, Spreading, and Migration Investigated with Deep Learning Platelet Morphometry

Jan Seifert,[1] Hendrik von Eysmondt,[1] Madhumita Chatterjee,[2] Meinrad Gawaz,[2] and Tilman E. Schäffer[1]*

[1] *Institute of Applied Physics, University of Tübingen, 72076 Tübingen, Germany*

[2] *Department of Cardiology and Angiology, University of Tübingen, 72076 Tübingen, Germany*

* Correspondence to:

Tilman E. Schäffer

Institute of Applied Physics

Eberhard Karls University Tübingen

Auf der Morgenstelle 10, 72076 Tübingen, Germany

E-mail: tilman.schaeffer@uni-tuebingen.de

Supplementary Figures

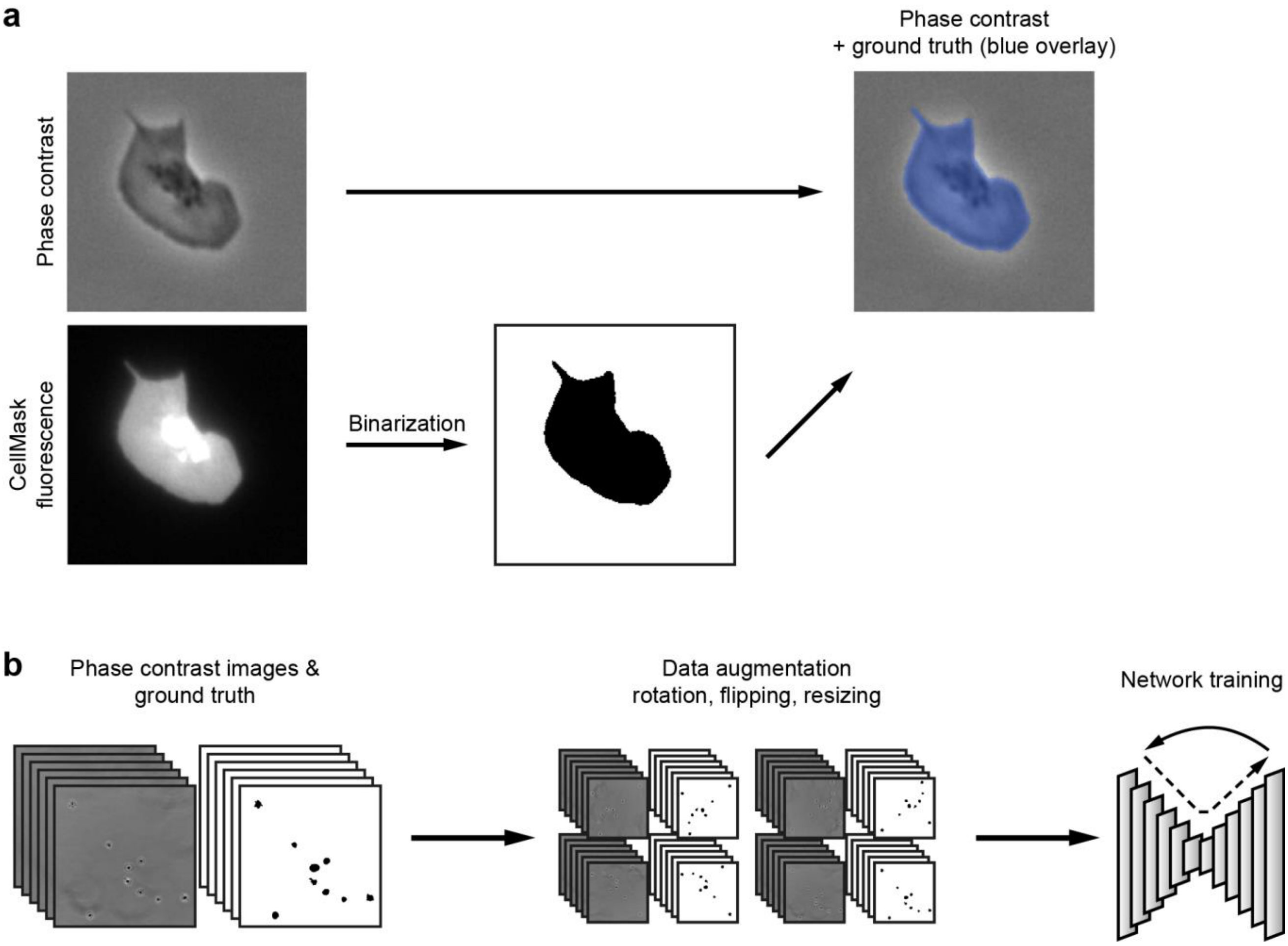

Supplementary Figure S1: Training of the CNN. (a) Generation of the binary ground truth: Pairs of phase contrast and fluorescence images of membrane-stained platelets were recorded. The fluorescence images were binarized and used as ground truth for neural network training. (b) Neural network training: Phase contrast images and ground truth were rotated, flipped, and resized by data augmentation to increase the data pool.

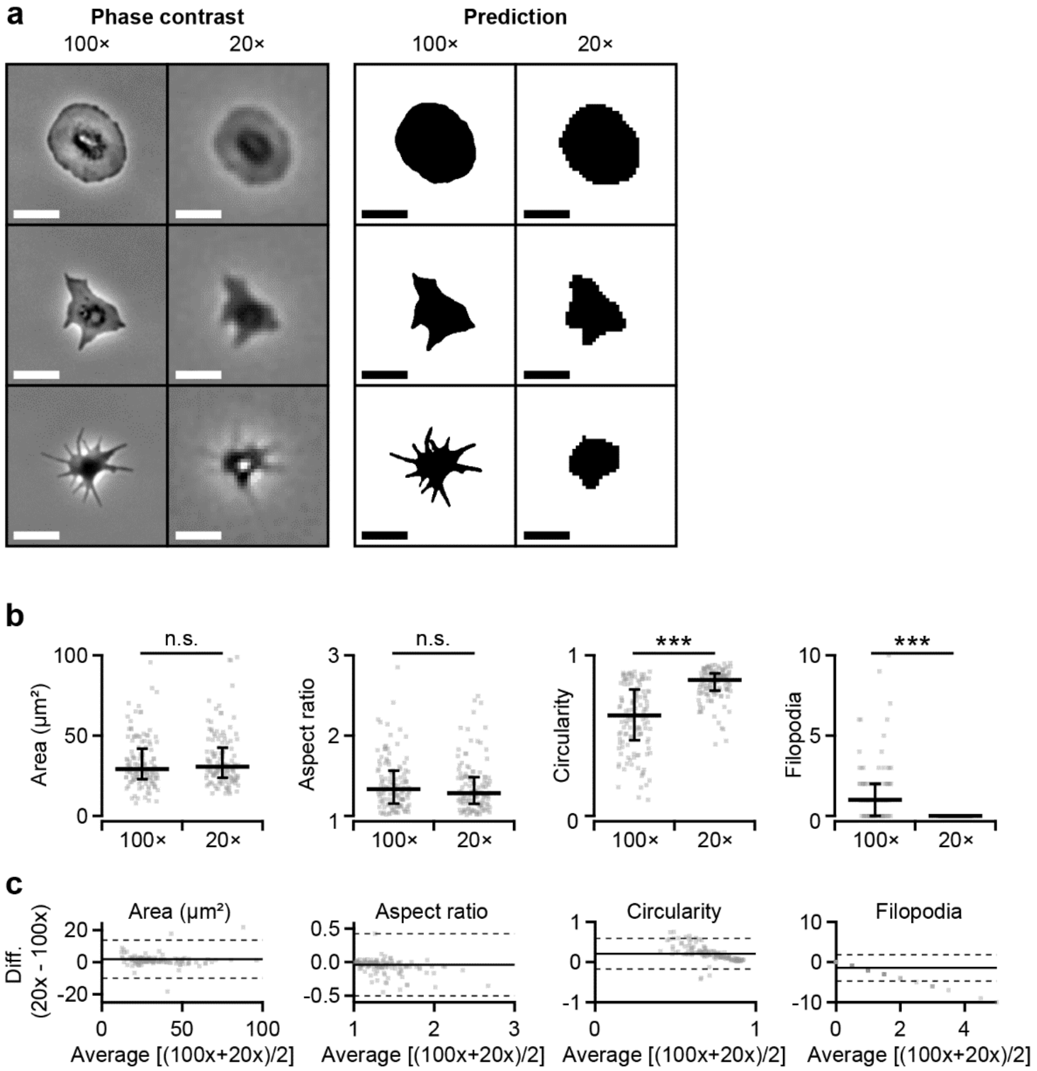

Supplementary Figure S2: Comparison of 100× and 20× magnification. (a) Phase contrast images of fixed platelets recorded with 100× and 20× magnification and the respective predictions by the CNNs. (b) Platelet shape parameters from the 100× and 20× CNNs. There is no significant difference for platelet area and aspect ratio. (c) Bland-Altman plots comparing the 100× and 20× shape parameters. The solid and dashed lines indicate the mean ± 1.96× standard deviation of the difference between the 100× and 20× shape parameters. *** indicates statistically significant difference ($P < 0.001$). Scale bars: 5 µm.

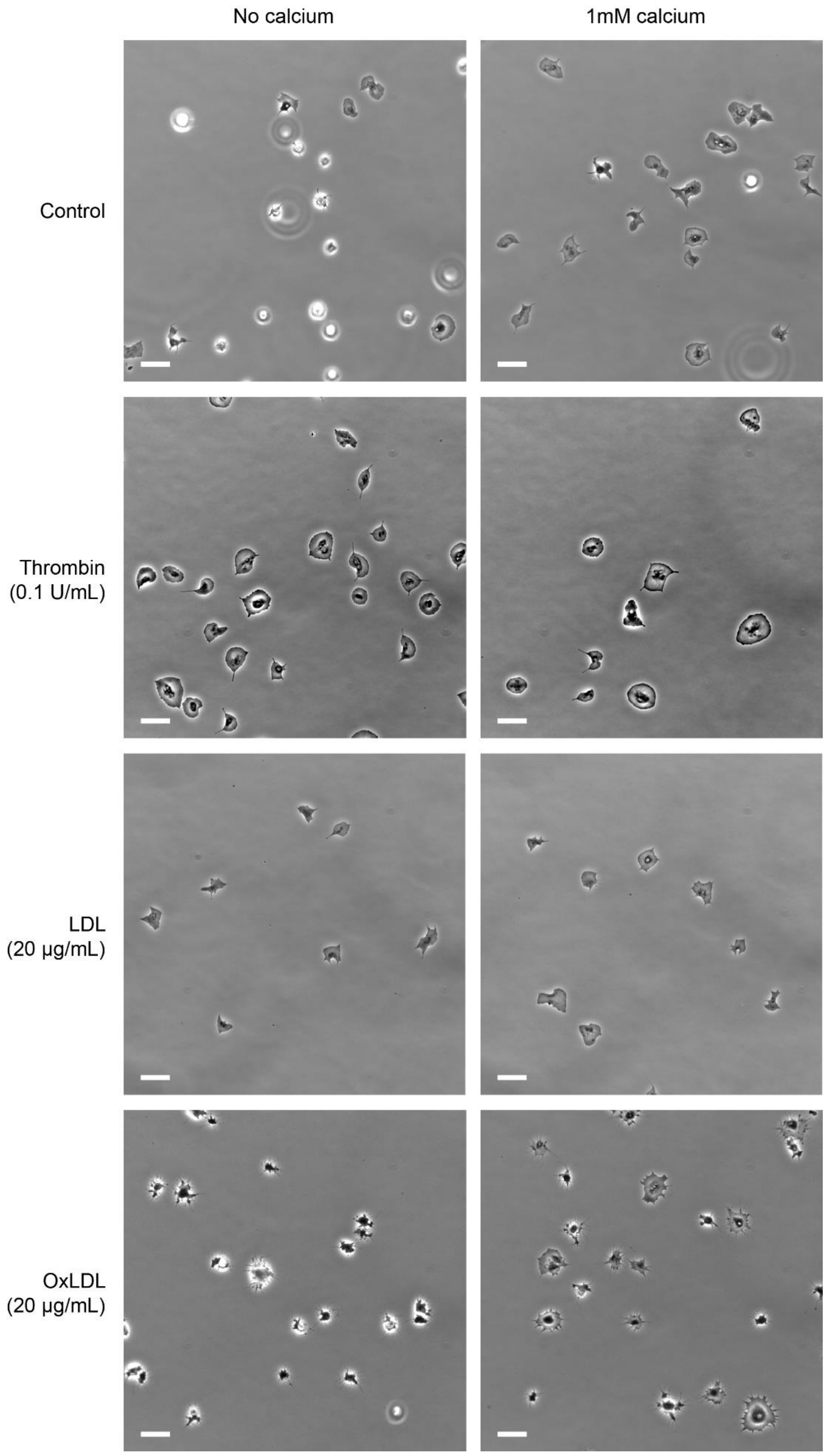
No calcium
1mM calcium
Control
Thrombin
(0.1 U/mL)
LDL
(20 µg/mL)
OxLDL
(20 µg/mL)

Supplementary Figure S3: Impact of extracellular calcium on platelet spreading. Full camera images showing multiple platelets spreading on 0.02 mg/mL fibrinogen without and with 1 mM extracellular calcium. Scale bars: 5 µm.

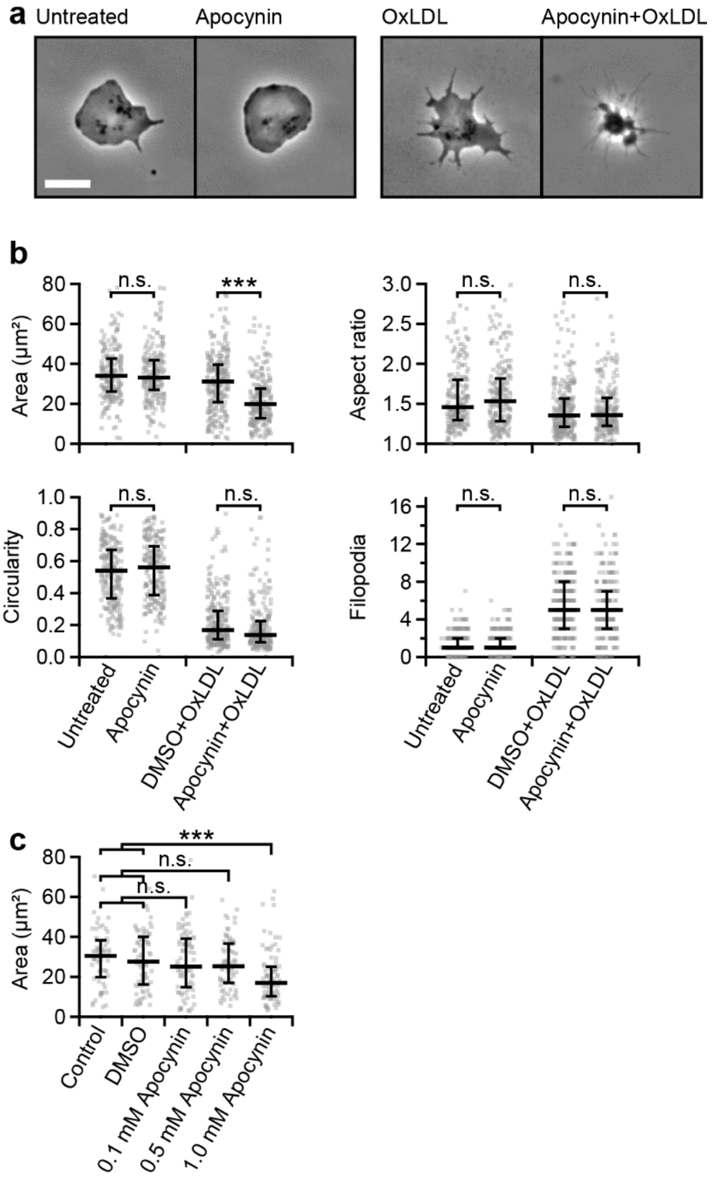

Supplementary Figure S4: Platelet shape and ROS generation. (a) Phase contrast images and (b) platelet shape parameters of untreated and oxLDL-treated platelets spread on 0.02 mg/mL fibrinogen for 30 min without and with pre-treatment with 1mM apocynin. (c) Spreading area of oxLDL-treated platelets pre-treated with DMSO or apocynin at the given concentration. *** indicates statistically significant difference (P < 0.001). Scale bars: 5 µm.

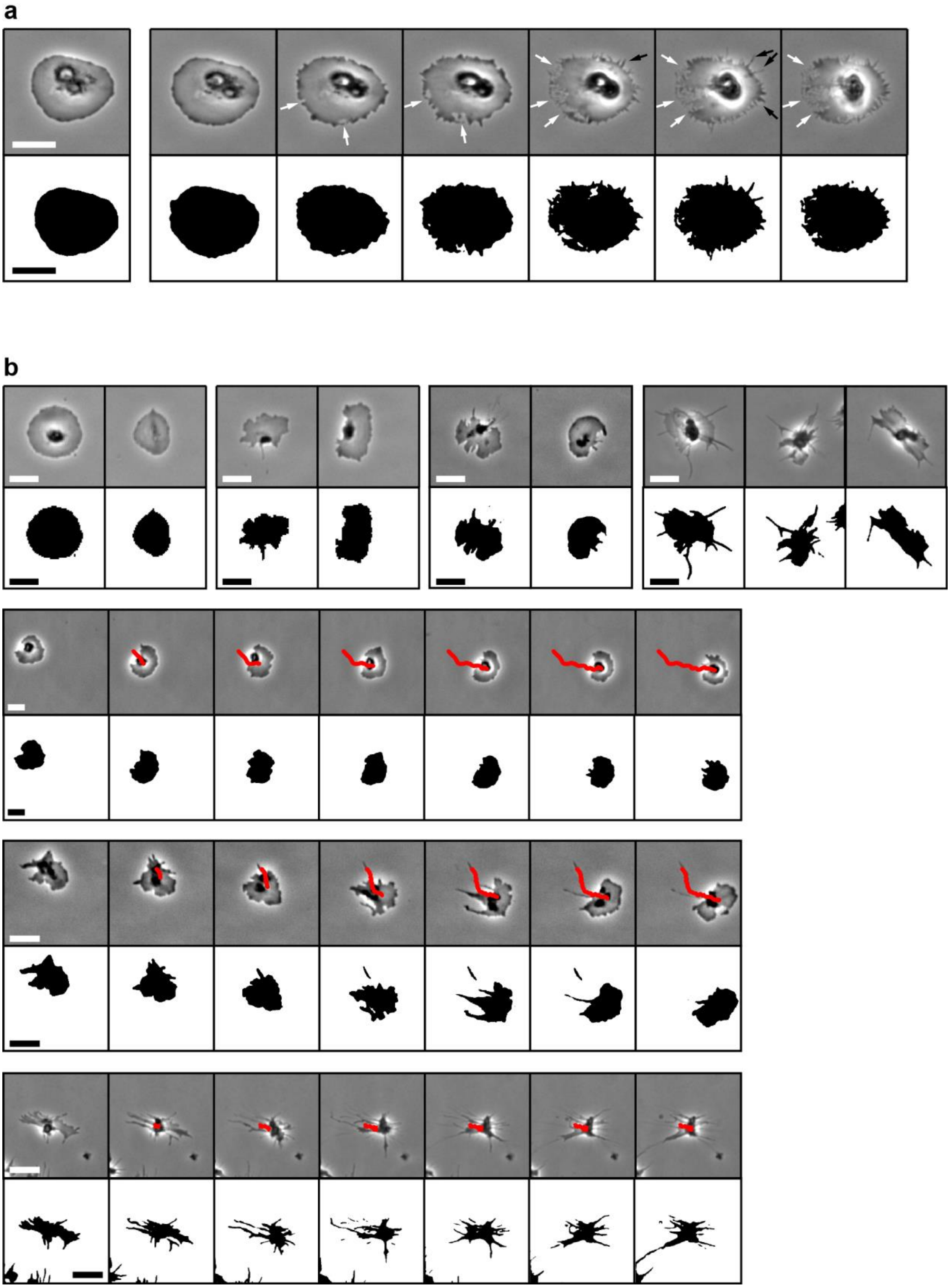

Supplementary Figure S5: CNN predictions. Phase contrast images and corresponding predictions from (a) Fig. 4 and (b) Fig. 5. Scale bars: 5 µm.

Supplementary Videos

Supplementary Video S1: Platelets with different treatments during spreading. Phase contrast image sequence and CNN predictions of untreated, thrombin-treated, LDL-treated, and oxLDL-treated platelets spreading on fibrinogen. Sequence is shown in Fig. 3a. Scale bars: 5 µm.

Supplementary Video S2: OxLDL-induced retraction of platelet lamellipodia. Phase contrast image sequence and CNN predictions of a platelet spread on fibrinogen during the addition of oxLDL. Sequence is shown in Fig. 4a. Scale bars: 5 µm.

Supplementary Video S3: Platelets with different treatments during haptotactic migration. Phase contrast image sequence and CNN predictions of untreated, LDL-treated, and oxLDL-treated platelets during haptotactic migration on fibrinogen. Sequence is shown in Fig. 5c. Scale bars: 5 µm.
